# The viral transactivator HBx protein exhibits a high potential for regulation via phosphorylation through an evolutionarily conserved mechanism

**DOI:** 10.1186/1750-9378-7-27

**Published:** 2012-10-18

**Authors:** Sergio Hernández, Mauricio Venegas, Javier Brahm, Rodrigo A Villanueva

**Affiliations:** 1 Laboratorio de Virus Hepatitis, Departamento de Ciencias Biológicas, Facultad de Ciencias Biológicas, Universidad Andrés Bello, Avda. República 217, 3er piso, Santiago 8370146, Chile; 2 Sección de Gastroenterología, Departamento de Medicina, Hospital Clínico Universidad de Chile, Avda. Santos Dumont 999, Independencia, Santiago 8340457, Chile

**Keywords:** Hepatitis B virus, Hepatitis B virus X protein (HBx), Posttranslational modification, Phosphorylation, O-β-glycosylation

## Abstract

**Background:**

Hepatitis B virus (HBV) encodes an oncogenic factor, HBx, which is a multifunctional protein that can induce dysfunctional regulation of signaling pathways, transcription, and cell cycle progression, among other processes, through interactions with target host factors. The subcellular localization of HBx is both cytoplasmic and nuclear. This dynamic distribution of HBx could be essential to the multiple roles of the protein at different stages during HBV infection. Transactivational functions of HBx may be exerted both in the nucleus, via interaction with host DNA-binding proteins, and in the cytoplasm, via signaling pathways. Although there have been many studies describing different pathways altered by HBx, and its innumerable binding partners, the molecular mechanism that regulates its different roles has been difficult to elucidate.

**Methods:**

In the current study, we took a bioinformatics approach to investigate whether the viral protein HBx might be regulated via phosphorylation by an evolutionarily conserved mechanism.

**Results:**

We found that the phylogenetically conserved residues Ser25 and Ser41 (both within the negative regulatory domain), and Thr81 (in the transactivation domain) are predicted to be phosphorylated. By molecular 3D modeling of HBx, we further show these residues are all predicted to be exposed on the surface of the protein, making them easily accesible to these types of modifications. Furthermore, we have also identified Yin Yang sites that might have the potential to be phosphorylated and O-β-GlcNAc interplay at the same residues.

**Conclusions:**

Thus, we propose that the different roles of HBx displayed in different subcellular locations might be regulated by an evolutionarily conserved mechanism of posttranslational modification, via phosphorylation.

## Background

HBx is the smallest gene of hepatitis B virus (HBV), and is highly conserved among all eight major genotypes (A to H) of the virus. The protein is common to all mammalian members of the *Hepadnaviridae* family, but is absent in the avian viruses. The primary amino acids sequence of HBx spans 154 residues, and is organized into two functional domains [1]. The N-terminal third is a negative regulatory domain, whereas the C-terminal two-thirds act as a transactivation domain. The negative regulatory domain has been mapped to the first fifty amino acid residues, including a Ser/Pro-rich (residues 21–50) dimerization region that is necessary for HBx dimerization [2]. The N-terminal region (residues 1–50) of HBx has been shown to be important for cellular transformation [3]. The transactivation domain has been mapped to the region between residues 53 and 142, and it has been shown that the negative regulatory domain is dispensable for this function, and actually represses HBx transactivation [4]. Additionally, the interaction of the transactivation domain of HBx with XAP-1/UVDDB and p53 has been mapped to regions 55–101, and 102–136, respectively [5,6].

The subcellular localization of HBx seems to be predominantly cytoplasmic, with a fractional nuclear distribution [7,8]. HBx is primarily localized in the nucleus at low expression levels but accumulates in the cytoplasm under conditions of elevated overexpression, indicating that the subcellular localization of the protein is influenced by its abundance [7,8]. Additionally, high levels of HBx lead to an abnormal mitochondrial distribution [9]. In the nucleus, HBx has been shown to transactivate a diverse array of viral and cellular promoters [10]. This ability suggests that HBx can upregulate the expression of viral genes by transactivating its own promoters. Given that HBx does not directly bind to DNA, its ability to activate transcription of host genes is thought to take place indirectly by interaction with nuclear transcription factors. Thus, HBx has been reported to associate with several components of the basal transcriptional machinery, such as TFIIB, TFIIH, and RBP5, a subunit of mammalian RNA polymerase [2]. It has also been demonstrated that HBx is able to bind transcription factors such as CREB, ATF-2, and AP-2 to modify their activities [11,12]. It was recently shown that HBx could interact and cooperate with CREB-binding protein (CBP)/p300 to synergistically enhance CREB activity [13]. Thus, the ability of HBx to interact with cellular factors may provide a mechanism for its role in transcriptional regulation.

Moreover, different cytoplasmic signal transduction cascades appear to be affected by HBx, including the stress-activated protein kinases/NH2-terminal-Jun kinase (SAPK/JNK), extracellular signal-regulated kinase (ERK), protein kinase B (PKB/Akt), Ras-Raf-mitogen-activated protein kinase (Ras-Raf-MAPK), the Janus kinase/STAT (JAK/STAT), and the adhesion kinase (FAK) and proline-rich tyrosine kinase 2 (Pyk2) [11,14]. Furthermore, the Wnt/β-catenin signaling pathway was also shown to be altered in the presence of HBx. Augmented expression of β-catenin occurs in 50–70% of human hepatocarcinoma (HCC) patients [15]. HBx has been shown to stabilize β-catenin independently of GSK3b in HCC cell lines [16,17]. Recently, the APC tumor suppressor was identified as a novel binding partner of HBx indicating its direct involvement in Wnt activation [18].

HBx has been found to be overexpressed in human HCC, and its overexpression in hepatocyte cultures, and transgenic mice results in a tumorigenic phenotype in some model systems [19,20]. It has been suggested that HBx interacts with the Ras-Raf-MAPK pathway to promote cell growth [21]. In addition, multiple pathways of DNA repair are also affected by HBx [22,23]. Moreover, it was found that both HBx and telomerase were highly expressed in hepatoma and liver cirrhosis tissues, and that HBx could upregulate the expression and activity of hTERT, the catalytic subunit of telomerase [24]. These findings suggest that HBx expression may play a role in the development of HCC by modulating telomerase activity.

Given the intricate molecular behavior of HBx including its subcellular distribution, the distinct roles displayed at different intracellular locations, and its many interacting partners, it has been difficult to elaborate a mechanism to explain the regulation of its functions. Since the primary sequence of the protein does not display any obvious localization signal, an as yet unexplored functional alternative is that the protein might be regulated by posttranslational modification.

Phosphorylation of proteins can regulate a viral protein′s subcellular localization, stability, biochemical and/or enzymatic activity, protein/nucleic acids interactions, and interactions with other cellular and viral partners, as previously reported for viruses such as rotavirus [25], hepatitis C virus [26], rabies virus [27], human papillomavirus [28], and varicella-zoster virus [29], among several others. For hepadnaviruses, phosphorylation is a fundamental event during genome replication. The phosphorylation status of the nucleocapsids has been shown to reflect the maturation stage of the viral particles [30,31]. On the other hand, there have been several previous reports investigating the phosphorylation of HBx. Since the protein is multifunctional and distributed in different subcellular locations, it is assumed that it could be regulated by phosphorylation, and it has been reported that its overexpression in different systems yielded phosphorylated HBx [32-34]. Moreover, recombinant HBx was found to be phosphorylated *in vitro* by protein kinase C (PKC), and mitogen-activated protein kinase (MAPK). Preliminary amino acid analysis showed that the phosphorylated residues could be serine [35]. Nevertheless, a functional role for the phosphorylation of HBx has not been yet reported.

In addition to phosphorylation by kinases, there is a different posttranslational modification, known as O-β-glycosylation that can take place in nearly all eukaryotic cells. Unlike phosphorylation modulated by host kinases, O-β-glycosylation is carried out by the activity of a single enzyme, O-linked N-acetylglucosamine (O-GlcNAc) transferase (OGT). O-GlcNAc is found on a wide range of proteins involved in virtually all cellular processes as well as various human diseases [36,37] including cancer [38]. In addition, O-GlcNAc can interplay with phosphorylation, which, for instance, modulates the stability and activity of p53 [39]. Furthermore, O*-*GlcNAc modifications have been found in several proteins from different viruses such as the cytomegalovirus basic phosphoprotein UL32 (pp150) [40], the adenovirus fiber protein [41], the baculovirus gp41 protein [42], and the nonstructural rotavirus NSP5 (NS26) protein [43] as well as the capsid protein from plum pox virus [44]. The biological relevance of these modifications is not yet known. However, intriguingly, several of these O-GlcNAc-modified viral proteins are also phosphoproteins, as in the cases of CMV pp150, and rotavirus NSP5. It remains to be determined if there is some interplay between the two types of modifications.

Phosphorylation and O-GlcNAc modifications on the same or neighboring Ser and Thr residues are known to occur in several nuclear and cytosolic proteins. This is known as the Yin Yang hypothesis, and the Ser and Thr residues involved in this interplay are considered Yin Yang sites. Typically, Yin Yang sites may compete for similar Ser or Thr residues or they might alter the substrate specificity of nearby sites by steric or electrostatic effects [45-48].

In the current article, our group has taken a bioinformatics approach to investigate whether the viral protein HBx might be regulated via phosphorylation by a phylogenetically conserved mechanism. The results obtained by this kind of approach should help in designing future new research lines, in order to expand our understanding on both the biology and virology of HBx protein.

## Methods

### HBV sequences

As previously reported, we have amplified, and sequenced several full-length HBV DNAs from human samples corresponding to isolates from genotype F [49]. The PCR amplified DNA corresponding to isolate HCUCH4 (accession number HM585186) was subcloned. One representative clone (Isolate 4.5) was further subjected to two-strand full-length sequencing, and has been shown to be able to replicate in cultured cells (data not shown). The sequence of the HBx protein from isolate 4.5 used in this study is “MAA RLC CQL DPA RDV LCL RPV SAE SCG RSL SGS LGA VSP PSP SAV PAD DGS HLS LRG LPV CSF SSA GPC ALR FTS ARR MET TVN APR SLP TVL HKR TLG LSG RSM TWI KEY IKD CVF KDW EEL GEE IRL KVF VLG GCR HKL VCS PAP CNF FTS A”. Further results about the functionality of clone isolate 4.5 will be published elsewhere.

**Figure 1:**
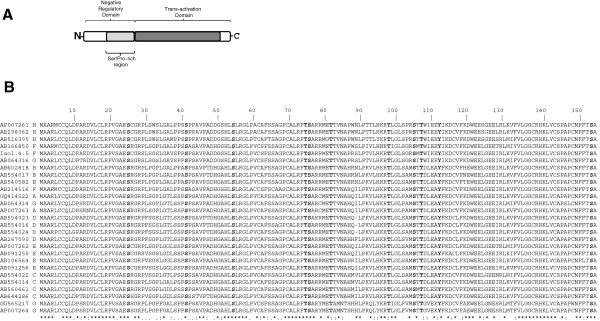
**Hepatitis B virus X protein (HBx). A**) Schematic representation of HBx domain organization. As shown, HBx is functionally organized into an N-terminal, and a C-terminal domain. The N-terminal third of HBx corresponds to the negative regulatory domain, which includes a Ser/Pro-rich region. The remaining C-terminal two-thirds of the protein comprises the transactivation domain. **B**) Multiple sequence alignment of HBx proteins from all main HBV genotypes using ClustalW. Accession numbers are indicated in the left column, followed by the HBV genotype of each isolate. Conserved Ser, Thr, and Tyr residues are shown in bold across the sequences. At the bottom of the alignment, the consensus sequence is marked by an asterisk, conserved substitutions by a double dot, and a semiconserved substitution by a single dot.

In the current study, HBx protein sequences from the eight major genotypes of HBV (A to H) were included, considering only full-length published isolates. Homology searches and sequence alignments (Figure 1B) were made with two protein sequences from genotype A (AP007263 and GQ414522), three sequences from genotype B (AB602818, AB554017, and AB540582), four sequences from genotype C (AB644286, AB560661, AB554022, and AB554014), four sequences from genotype D (AB554023, AB267090, AB554016, and AB554024), four sequences from genotype E (AP007262, AB106564, AB091255, and AB091256), four sequences from genotype G (GU565217, AB064316, AP007264, and AB064314), three sequences from genotype H (AB516395, AP007261, and AB298362), and two sequences from genotype F (AB166850, and AB214516). The sequence of HBx from HCUCH4 (ADV59932) differs from that of isolate 4.5 at amino acid residue 109 because of an E - > K substitution, respectively. Homology analysis, and multiple alignments of protein sequences were carried out with ClustalW at the Pôle Bioinformatique Lyonnais web site (pbil.univ-lyon1.fr/).

### Post-translational modification predictions

For all the prediction analyses, we used the sequence of the HBx protein corresponding to clone isolate 4.5 as a reference.

To analyze the phosphorylation potential of the HBx protein, we utilized several servers on the web such as NetPhos 2.0, DISPHOS 1.3, PPRED, and Phos3D [50-53]. These are neural networks-based programs that predict potential sites of serine, threonine, and tyrosine phosphorylation in polypeptides (Table 1). The minimum threshold value used to predict phosphorylation is 0.5 on both NetPhos 2.0, and DISPHOS. On PPRED, the analysis was made with default parameters. The Phos3D results were obtained considering a decision value > 0 as a positive prediction.

**Table 1:** Bioinformatics tools used for prediction

**Name**	**Technique**	**Residues**	**Reference**	**Website**
**Phosphorylation**				
NetPhos 2.0	ANN	9-33	[51]	http://cbs.dtu.dk/services/NetPhos
DISPHOS 1.3	LR	25	[52]	http://dabi.temple.edu/disphos
PPRED	PSSM, SVM	7-15	[54]	http://ashiskb.info/reserach/ppred
Phos3D	SVM	13	[53]	http://phos3d.mpimp-golm.mpg.de
**Kinases**				
NetPhosK 1.0	ANN	9-33	[55]	http://cbs.dtu.dk/services/NetPhosK
KinasePhos	SVM	9	[56]	http://kinasephos2.mbc.nctu.edu.tw
PPSP	BP	9	[57]	http://ppsp.biocuckoo.org
GPS 2.1	PSSM, GA	3-31	[58]	http://gps.biocuckoo.org

Techniques: *ANN*: Artificial neural network; *LR*: Logistic regression; *SVM*: Support vector machine;

*PSSM*: Position-specific scoring matrix; *BP*: Bayesian probability; *GA:* Genetic algorithm.

Specific kinases for phosphorylation positions in HBx were also predicted by NetPhosK 1.0, KinasePhos, PPSP, and GPS 2.1 [54-57]. These servers predict kinase specific acceptor substrates including serine, threonine, and tyrosine residues.

Potential O-β-GlcNAc modification sites were predicted by the server. This program can predict potential phosphorylation sites as well as predict Yin Yang sites with a highly uneven threshold that is adjusted in accordance with amino acid surface accessibility [58].

### Protein structure analysis

Since there is no template model of HBx available, we designed an ab-initio model using the software I-TASSER (zhanglab.ccmb.med.umich.edu/I-TASSER/; [59]). Data in sequence form was uploaded to the server. The model with the highest C-score was selected for further analyses. To view, and analyze the 3D structure of HBx, both RasMol v. 2.7.5.2., and MVP (Macromolecular Visualization and Processing, v. 1.0) software were used. To assess whether the predicted serine and threonine residues have surface accessibility for posttranslational modifications, NetSurfP was utilized [60].

## Results

The hepatitis B virus HBx protein includes a primary sequence of 154 amino acid residues, and has been dissected into two functional regions, as shown in Figure 1A. The N-terminal third (the first fifty amino acid residues) is a negative regulatory domain, whereas the C-terminal two-thirds is a transactivation domain. The negative regulatory domain includes a Ser/Pro-rich (residues 21–50) dimerization region, whereas the transactivation domain has been mapped to the region between residues 53 and 142 [1,2]. Thus, since the protein might be subjected to complex regulation to display all its roles within the nucleus as well as in the cytoplasm, we searched for a phylogenetically conserved mechanism of regulation such as phosphorylation.

The HBx protein from clone isolate 4.5 was aligned together with 26 other HBx protein sequences including all main HBV genotypes (genotypes A-H). Conserved serine, threonine, and tyrosine residues were determined across all main genotypes, as shown in Figure 1B. It is clear from the figure that serines 25, 41, 54, 75, 104, and 153; threonines 74, 81, 97, and 106; tyrosine 111 are all conserved; therefore each of them might be a target for an evolutionarily conserved mechanism of regulation, via phosphorylation. Interestingly, both serine residues 25 and 41 are included in the negative regulatory domain, and within the dimerization region of HBx, whereas all the other conserved positions are included in the transactivation domain of the protein.

To predict potential phosphorylation sites among the conserved serine, threonine, and tyrosine residues of HBx, we used several servers available on the web (Table 1). Prediction results are shown on Table 2. The combined results from NetPhos 2.0 and DISPHOS 1.3 indicated that the serine residues, the prediction of the phosphorylation scores was Ser75 > Ser41 > Ser25. Ser54 presented a score close to threshold. However, considering the spatial context, Phos3D predicted that only Ser25 and Ser41 would be phosphorylated. These data were further confirmed by NetSurfP 1.1, which predicted that both Ser25 and Ser41 are exposed residues. As indicated above, both Ser25 and Ser41 are located within the Ser/Pro-rich region in the negative regulatory domain of HBx. Among HBx threonine residues, both NetPhos 2.0 and DISPHOS 1.3 predicted the phosphorylation of Thr81. These data were further confirmed by Phos3D, and according to the analysis of the NetSurfP 1.1, this residue is expected to be exposed. Interestingly, among the conserved threonine residues in HBx, phosphorylation of Thr81 will take place within the transactivation domain of the protein. The conserved Tyr111 scored below to the threshold, and furthermore this position is expected to be buried, and is, therefore, not predicted to be phosphorylated.

**Table 2:** Phosphorylation prediction on HBx

**Conserved residues**	**NetPhos 2.0**	**DISPHOS 1.3**	**PPRED**	**Phos3D**	**NetSurfP 1.1**	**NetPhosK 1.0**	**KinasePhos**	**PPSP**	**GPS 2.1**	**YinOYang 1.2**
**Serines**										
S25	0.961	0.762	―	+	E	CKI	―	―	TKL MLK ILK	―
S41	0.983	0.930	+	+	E	P38MAPK GSK3 cdk5	cdc2 MAPK	KIS	CMGC MAPK ERK	+
S54	0.652	0.672	―	―	E	CKI PKC	―	MAPKKK	―	―
S75	0.984	0.672	―	―	B	―	―	―	AGC PKC CAMK	―
S153	0.005	0.187	―	―	E	―	―	―	AGC GRK TKL	―
**Threonines**										
T74	0.147	0.115	―	―	B	PKC	―	PKR	―	―
T81	0.947	0.823	+	+	E	―	PKA	MAPKKK	CAMK DAPK LKB	+
T97	0.017	0.085	―	―	E	PKG	PKA	ILK	TLK MLK ILK	―
T106	0.524	0.113	―	―	E	―	―	―	AGC SGK CAMKL	―
**Tyrosines**										
Y111	0.031	0.062	―	―	B	―	―	―	TK PDFGR Eph	―

Netphos: threshold > 0.5; DISPHOS: threshold >0.5; PPRED, Phos3D: parameters as default; NetSufP: E: exposed surface;

*B*: buried surface; O-β-GlcNAc: threshold as default.

Different kinases may be implicated in the phosphorylation of serine, threonine and tyrosine residues. Several protein kinases are also involved in phosphorylating two or more kinds of residues. The kinases predicted to be involved in phosphorylation of conserved residues of HBx are shown in Table 2. As shown, several kinases are predicted to phosphorylate the same phosphorylation site since the local amino acid sequence around the phosphoacceptor residue can be recognized by them.

**Figure 2:**
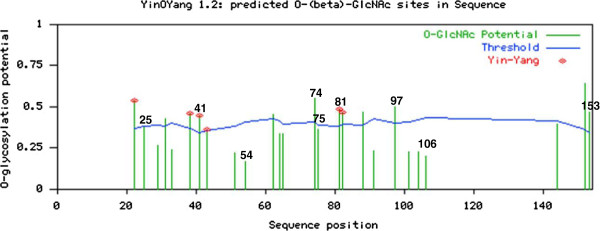
**Predicted O-glycosylation and Yin Yang sites on the HBx protein. **The O-β-GlcNAc modification potential of each Ser, and Thr residues is shown by green vertical lines, and the light blue horizontal wavy line indicates the threshold for modification potential. The Yin Yang sites that were positively predicted are shown by red asterisks at the top. The profile was obtained with server YinOYang 1.2, using the HBx sequence of our HBV isolate 4.5 as a reference. The numbers at the top of lines of O-glycosylation potential indicate the positions of conserved HBx Ser, Thr or Tyr residues. As shown, Ser25 represents a FN Yin Yang site.

The prediction results for O-linked glycosylation sites showed that HBx exhibits the potential for O-β-GlcNAc modification, as shown in Figure 2. The conserved HBx Ser and Thr residues predicted to be O-β-GlcNAc modified are Ser41, Ser153, Thr74, Thr81, and Thr97. On the other hand, the conserved HBx Ser, and Thr residues predicted to be positive sites for Yin Yang modification are Ser41 and Thr81, as indicated in Figure 2. In addition to the positive Yin Yang sites, in several instances, the same Ser and Thr residues show a very high potential for phosphorylation, and also show a potential very close to the specific threshold value for O-β-glycosylation as predicted by existing methods. Such sites are termed false-negative (FN) Yin Yang sites, when they are evolutionary conserved, as on these sites the OGT enzyme and kinases may have similar accessibility for inducing posttranslational modifications of interest [61]. Following this criteria, we identified the conserved Ser25 as a FN Yin Yang site (Figure 2).

**Figure 3:**
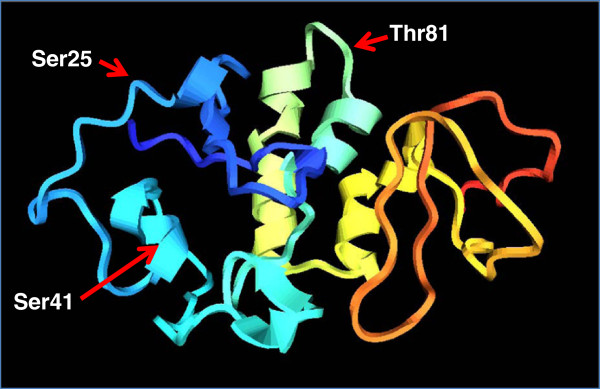
**Homology 3D modeling of HBx. **The HBx sequence of our isolate 4.5 used as a reference was submitted to server I-Tasser for protein structure prediction. Out of five models developed by the server, we selected the model with the highest C-value. To view and analyze HBx 3D structure, both RasMol v. 2.7.5.2., and MVP (Macromolecular Visualization and Processing, v. 1.0) software were used. The positions of Ser25, Ser41, and Thr81 are shown in the model.

In order to analyze the location of the conserved Ser, and Thr residues predicted to be phosphorylated within HBx, we drew a 3D model of the protein. We also assessed the possible surface accessibility of HBx for these posttranslational modifications. In the model shown in Figure 3, and consistent with the results shown above, we found that Ser25, Ser41, and Thr81 are all exposed residues within the 3D model of HBx. This information depicts that these Ser and Thr positions exhibit ready access to these types of modifications.

## Discussion

Chronic hepatitis B virus infection has been strongly associated with the development of hepatocellular carcinoma. HBV encodes an oncogenic factor, HBx, which is a multifunctional regulator that modulates signal transduction pathways, gene transcription, cell cycle progression, protein degradation, apoptosis, and genetic stability through direct and indirect interactions with target cell factors [2]. The subcellular localization of HBx is primarily cytoplasmic, with a small fraction in the nucleus [7,8]. The dynamic allocation of HBx could be important for the multiple roles of this protein at different stages in the HBV life cycle. Transactivational functions of HBx may be exerted both in the nucleus, via interactions with host DNA-binding proteins, and in the cytoplasm, via signaling pathways [2]. Although there have been many studies describing different pathways altered by HBx, and its innumerable binding partners, the molecular mechanism that regulates its different roles has been difficult to elucidate.

In order to analyze whether HBx might be regulated by a mechanism that involves a posttranslational modification such as phosphorylation, we took a bioinformatics approach, searching for an evolutionarily conserved mechanism of regulation. In strong support of our analysis, earlier observations have indicated that HBx was phosphorylated upon overexpression of the protein in different systems [32-35]. In the current study, to predict the likehood of phosphorylation on phylogenetically conserved Ser, Thr or Tyr HBx residues, we combined the utilization of multiple neural network-based softwares that predict potential phosphorylation sites with the use of several servers that predict kinase-specific acceptor substrates together with molecular 3D modeling of HBx. Our analysis indicated that Ser25, Ser41, and Thr81 exhibit high potential for phosphorylation via a conserved mechanism. These positions were all predicted to have high potentials for phosphorylation by servers such as NetPhos 2.0, and DISPHOS 1.3, and the results were confirmed by servers such as PPRED. Predictions of phosphorylation on Ser25, Ser41, and Thr81 were further verified by the Phos3D program, which considers information in a spatial context. NetSurfP analysis which predicts surface accessibility, and secondary structure within an amino acid sequence indicated that all three residues were exposed.

In addition to phosphorylation by kinases, we also checked predictions of O-β-glycosylation sites on HBx. It has been demonstrated that phosphorylation and O-GlcNAc modifications on the same or neighboring Ser and Thr residues occur in several nuclear and cytoplasmic proteins, and phosphorylation and O-β-GlcNAc modifications are thought to establish an interplay in what are considered Yin Yang sites [45-48]. Yin Yang sites may compete for similar Ser or Thr residues or they might alter the substrate specificities of nearby positions by steric or electrostatic effects [48]. Ser41 and Thr81 are conserved HBx Ser and Thr residues predicted to be positive sites for Yin Yang modification. In addition, we found that Ser25 was predicted to be a false-negative (FN) Yin Yang sites. Ser25 was not predicted to be O-β-GlcNAc, although it was close to threshold value, and showed very high potential for phosphorylation. In addition, we found that the conserved Ser25, Ser41, and Thr81 are all exposed residues within the predicted 3D model of HBx. This information carries the implication that these Ser and Thr positions are readily accessible to these modifications.

Together, our results lead us to speculate that the conserved HBx residues Ser25, Ser41, and Thr81 may be potential phosphorylation sites that can regulate the roles of the HBx protein. Interestingly, both the Ser25 and Ser41 residues are located in the N-terminal, negative regulatory domain, and within the Ser/Pro-rich dimerization region, whereas Thr81 is located within the transactivation, C-terminal domain of HBx. Whether phosphorylation of each of these residues actually activates/represses several HBx protein functions remains to be experimentally determined, for example by targeted mutagenesis.

Phosphorylation events are one way in which animal viruses can make use of cellular signaling pathways for their own benefits, and the literature is well provided with different kinds of examples. In the case of HIV-1, the p6 protein contains the late domain involved in virus budding. This domain was determined to be phosphorylated by different kinases [62]. Further analysis identified a specific residue, Thr23, which is phosphorylated by MAPK, and ERK-2, both *in vitro* and *in vivo*[63]. On the other hand, the rubella virus capsid protein is phosphorylated at various sites by unknown kinases, and these phosphorylations are important for optimal viral replication [64,65].

A number of cellular enzymes that have been implicated in nucleic acid metabolism, such as the DNA-dependent RNA polymerases I and II [66,67], DNA polymerase α [68], and DNA topoisomerase IIα [69], are actually phosphoproteins, whose functions are regulated by phosphorylation, via kinases. Several viral enzymes are also regulated by phosphorylation. The RNA polymerase of the dengue virus (type 2) is phosphorylated on a serine residue by casein kinase II, and this phosphorylation regulates the interaction of the polymerase with other viral proteins, and the function of the viral replicase complex [70]. Furthermore, a phosphorylation in the N-terminal region of the RNA polymerase 2a protein from cucumber mosaic virus (CMV) by a 60 kDa protein kinase was shown to inhibit the interaction of the 2a polymerase with the 1a protein, whose interaction is essential for the genome replication of this plant virus [71].

Recently, Khattar et al. published a report where the Ser31 residue of HBx was shown to be phosphorylated by Akt 1 kinase [72]. The interaction between HBx and Akt was essential for Akt signaling, and it enhanced the oncogenic potential of HBx. However, a simple examination of HBx amino acid sequences from different isolates indicates that HBx Ser31 is not a conserved residue. Whereas HBV isolates from genotypes A, C, D, E and H all contain the Ser31 residue, some isolates from genotypes F and G do not, and isolates from genotype B all contain a Pro31 residue. It is clear from this analysis that phosphorylation of Ser31 does not reflect a conserved mechanism of regulation of HBx, and further, it might indicate why some HBV isolates are more oncogenic or pathogenic than others.

## Conclusions

Taken together, our analyses and results indicated that the roles of HBx might be regulated by an evolutionarily conserved mechanism of posttranslational modification, via phosphorylation of Ser25, Ser41, and/or Thr81.

### Future perspectives

Bioinformatics offers different ways for looking at problems in biology, analyzing data to generate new knowledge that might be useful in diverse fields such as biological pathway regulation, and drug design, among others. In the case of the viral HBx protein, utilizing bioinformatics specific software, we have found several amino acid residues that exhibit a clear potential for being phosphorylated by cellular protein kinases. However, the information presented in the current study will need to be experimentally validated both for individual HBx proteins within cells, and HBx regulation in the context of an HBV infection. We hope our study will contribute to further understanding the regulation of the viral HBx protein.

## Abbreviations

HBV: Hepatitis B virus; HBx: Hepatitis B virus protein X.

## Competing interests

The authors declare that they have no competing interests.

## Authors' contributions

RAV designed the study, and obtained the data. SH, MV, JB, and RAV analyzed the data, and wrote the paper. RAV carried out final editing and submission. “All authors read and approved the final manuscript.”
